# Cerebral Sinovenous Thrombosis

**DOI:** 10.3389/fped.2017.00163

**Published:** 2017-07-27

**Authors:** Rebecca Ichord

**Affiliations:** ^1^Department of Neurology and Pediatrics, Children’s Hospital of Philadelphia, Perelman School of Medicine of the University of Pennsylvania, Philadelphia, PA, United States

**Keywords:** cerebrovascular disorders, stroke, thrombosis, neonatal, childhood

## Abstract

Cerebral sinovenous thrombosis (CSVT) is a rare but serious cerebrovascular disorder affecting children from the newborn period through childhood and adolescence. The incidence is estimated at 0.6/100,000/year, with 30–50% occurring in newborns. Causes are diverse and are highly age dependent. Acute systemic illness is the dominant risk factor among newborns. In childhood CSVT, acute infections of the head and neck such as mastoiditis are most common, followed by chronic underlying diseases such as nephrotic syndrome, cancer, and inflammatory bowel disease. Signs and symptoms are also age related. Seizures and altered mental status are the commonest manifestations in newborns. Headache, vomiting, and lethargy, sometimes with 6th nerve palsy, are the most common symptoms in children and adolescents. Recent multicenter cohort studies from North America and Europe have provided updated information on risk factors, clinical presentations, treatment practices, and outcomes. While systemic anticoagulation is the most common specific treatment used, there are wide variations and many uncertainties even among experts concerning best practice. The treatment dilemma is especially pronounced for neonatal CSVT. This is due in part to the higher prevalence of intracranial hemorrhage among newborns on the one hand, and the clear evidence that newborns suffer greater long-term neurologic morbidity on the other hand. With the advent of widespread availability and acceptance of acute endovascular therapy for arterial ischemic stroke, there is renewed interest in this therapy for children with CSVT. Limited published evidence exists regarding the benefits and risks of these invasive therapies. Therefore, the authors of current guidelines advise reserving this therapy for children with progressive and severe disease who have failed optimal medical management. As research focused on childhood cerebrovascular disease continues to grow rapidly, the future prospects for improving knowledge about this disorder should be good.

## Definitions, Incidence, and Spectrum of Disease

**Figure 1:**
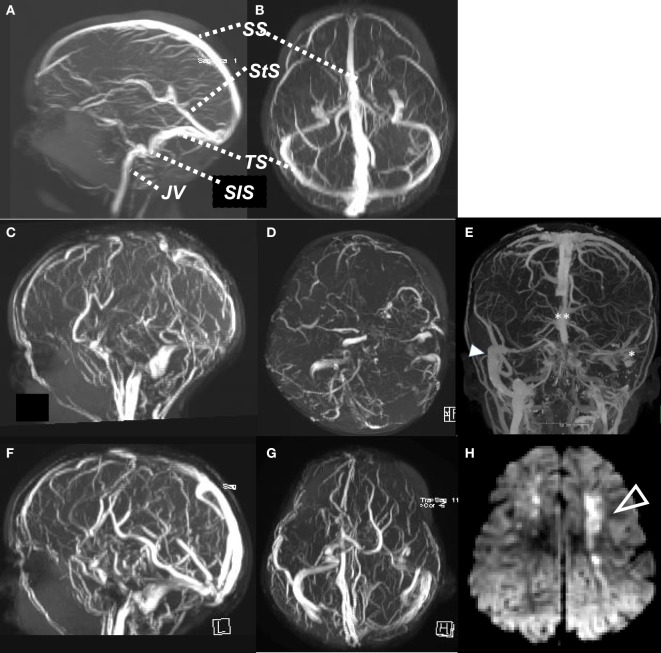
Normal MR venogram, sagittal view **(A)**, axial view **(B)**. Case of cerebral sinovenous thrombosis, acute MR venogram sagittal **(C)** and axial **(D)**, showing absent flow signal in SS, both TS and StS, with ischemic change (arrow) in cortical white matter on DWI **(H)**. Computed tomography venogram **(E)** of child with mastoiditis and occlusive thrombus of left SiS and TS (*) and distal SS (**), compared to patent right SiS and TS (arrow). MR venogram 6 months later **(F,G)**, with recanalization of SS, StS, and TS. SS, sagittal sinus; StS, straight sinus; TS, transverse sinus; SiS, sigmoid sinus; JV, jugular vein.

Cerebral sinovenous thrombosis (CSVT) encompasses a spectrum of disorders involving thrombosis of the cerebral venous system. The incidence in Europe and North America is estimated at 0.6 per 100,000 per year in childhood, with a male predominance (60–70%), and neonates accounting for 30–50% of cases (1, 2). The cerebral venous system is composed of a network of cortical, medullary, and deep veins which drain into dural venous sinuses. These comprise the superficial dural sinuses (sagittal, transverse, and sigmoid) and the deep venous system (straight sinus, vein of Galen) (see Figure 1). Thrombosis in the cerebral venous system impedes venous outflow, resulting in increased central venous pressure, which in turn causes intracranial hypertension. In some cases, this leads to cerebral ischemia, which may evolve to infarction, often hemorrhagic. In the most severe cases, diffuse cerebral edema and widespread infarction and hemorrhage may result in permanent neurologic disability, or herniation and death. Risk factors are diverse and are related to age, as well as the presence of acute or chronic illnesses, and thrombophilias (Table 1). The long-term outcome of CSVT in children is variable and depends on the age of incident disease, comorbid diseases, and presence of acute complications (Table 2). Neonates in general have a greater risk of poor outcomes, including motor and cognitive impairments and, notably, epilepsy. Children with CSVT mostly do well and make a full recovery. Recurrence rates are low (<10%).

**Table 1:** Risk factors for cerebral sinovenous thrombosis (CSVT) in pediatric cohort studies.

Risk factor or inciting illness	Prevalence of risk factor (%)^a^						
	Ichord et al. (3) (IPSS)	Jordan et al. (4) (IPSS)	Grunt et al. (1) (Swiss)		Moharir et al. (5, 6) (Canadian)		Berfelo et al. (7) (Netherlands)
				
	*N* = 170 children	*N* = 84 neonates	*N* = 42 children	*N* = 21 neonates	*N* = 79 children	*N* = 83 neonates	*N* = 52 neonates
Acute systemic illness^b^	46	63	–	80	28	37	23
Acute head/neck infection or meningitis^c^	54	–	63	5	47	34	–
Prothrombotic state^d^	20	10	42	42	84/25^h^	67/21^h^	24
Hematologic disorder^e^	10	–	2	–	n/r^i^	n/r	–
Cancer	12	–	14	–	n/r	n/r	–
Immunologic disease^f^	4	–	14	–	n/r	n/r	–
Cardiac disease	2	13	9	–	n/r	n/r	2
Other chronic disease^g^	5	–	5	–	53^i^	18^i^	–

*IPSS, International Pediatric Stroke Study*.

*^a^Risk factors, note that many patients have multiple risk factors*.

*^b^Acute systemic illness—sepsis, respiratory failure, hypoxia/ischemia, gastroenteritis, dehydration*.

*^c^Acute head/neck infection—mastoiditis, sinusitis*.

*^d^Prothrombotic state—deficiencies in protein C, protein S, antithrombin III, factor V Leiden or prothrombin gene mutation, homocysteine elevation, lipoprotein a elevation, anticardiolipin antibodies, lupus anticoagulant*.

*^e^Hematologic disorder—anemia, hemoglobinopathies*.

*^f^Immunologic disease—lupus, Behcet’s disease*.

*^g^Other chronic disease—diabetes, nephrotic syndrome, inflammatory bowel disease*.

*^h^At diagnosis/persistent on f/u testing*.

^i^n/r, not specifically reported, pooled into “chronic systemic disease.”

**Table 2:** Anticoagulation treatment practices and outcomes.

Study	Population studied	*N*, median age, gender	AC Rx (%)	Outcome (%)		
				Mortality	Adverse outcomes	Epilepsy
Sébire et al. (8)	Europe 1993–2002	42 children, 5.7 years, 64% male	42	12	33	7
Mallick et al. (2)	United Kingdom 1997–2005	21 children, 7.1 years, 47% male	100, tPA 4/21	9	48	n/a
Mohariret al. (5)	Canada 1992–2005	83 neonates, 79 children, 5.5 years, 66% male	Neonates 35, children 71	Neonates 6, children 0	Neonates 59, children 37	n/a
Grunt et al. (1)	Switzerland 2000–2008	21 neonates, 67% male, 44 children, 8.7 years, 68% male	Neonates 33, children 90	9^a^	Neonates 38, children 4	Neonates 38, children 0
Berfelo et al. (7)	Netherlands 1999–2009	52 neonates, 75% male	42	20	55	n/a
Jordan et al. (4)	IPSS 2003–2007	84 neonates, 74% male	52	2	46^b^	n/a
Ichord et al. (3)	IPSS 2003–2007	170 children, 7.2 years, 60% male	83	4	52^b^	n/a

*IPSS, International Pediatric Stroke Study; AC Rx, anticoagulation treatment; tPA, systemic thrombolysis*.

*^a^Deaths not directly related to CSVT*.

*^b^Abnormal neurologic exam at hospital discharge; long-term outcome unknown*.

## Causes and Risk Factors

Causes and risk factors for CSVT are age dependent and highly variable (3, 4, 8, 9). They can best be understood in the context of Virchow’s triad: slowing or stasis of blood flow, injury or disruption of the vessel wall, and perturbation of the components of blood affecting clot formation and lysis. Frequently there are multiple coexisting inciting conditions and underlying risk factors. Among newborns, acute systemic illness, infection, and fluid/electrolyte disturbances are most common. Among previously healthy children, CSVT most often occurs in the setting of head/neck infections, acute illness with dehydration and iron deficiency anemia. Chronic illnesses that predispose children to CSVT include inflammatory bowel disease, cancer, autoimmune disorders, and chronic kidney disease, among others. Recently published multicenter cohort studies have described the most common risk factors and underlying conditions as shown in Table 1. Consistent findings across multiple cohort studies are that neonates have distinct risk factor profiles compared to children. Among neonates, exposure to perinatal stress (hypoxia/ischemia, difficult birth), and acute systemic illness such as sepsis, pneumonia, and respiratory distress syndrome are the leading predisposing and comorbid conditions. In childhood, previously healthy children develop CSVT mainly in the setting of acute treatable infections, in particular head and neck infections such as mastoiditis and sinusitis. Among children with chronic disease, certain diseases are particularly associated with a risk of CSVT due to disturbed regulation of coagulation or systemic circulation. These include complex congenital heart disease, inflammatory bowel disease, Behcet’s syndrome, nephrotic syndrome, and leukemia, especially during treatment with l-asparaginase.

Abnormal levels of prothrombotic factors are common in neonates and children. Some abnormalities are inherited, while others are acquired and may be transient. There is controversy as to whether some of these may be epiphenomena, coincidental vs causal in nature. Large scale case–control studies in adults with CSVT provide good evidence that certain prothrombotic risk factors do occur with a prevalence estimated at 30–35%, and indeed contribute to the causation of CSVT, often in combination with other inciting or comorbid diseases (10, 11). The prothrombotic factors most studied and shown to increase the risk of CSVT include deficiencies of protein C, protein S, and antithrombin III; mutations in the factor V Leiden and prothrombin genes; elevated blood levels of homocysteine; elevated anticardiolipin antibodies and lupus anticoagulant; elevated levels of lipoprotein a. Pediatric cohort studies and case–control studies report somewhat higher prevalence rates of prothrombotic factors than in adults, ranging from 25 to 60% and confirm associations with similar risk factors as in adults (12, 13). The Canadian cohort study evaluated the prevalence of prothrombotic factors acutely and again at follow-up, showing that many of the abnormalities in factor levels detected acutely normalized on follow-up testing (5). Interpretation of such abnormalities is especially complex among newborns, where the levels of endogenous fibrinolytic factors such as protein C and S are normally low based on age, or may be decreased secondarily by the acute illness. These observations mean that results of cohort studies must be interpreted with caution and can only be understood in the context of age-specific norms, the state of the child at the time of testing, and results of follow-up testing (14).

## Clinical Signs and Symptoms

Clinical signs and symptoms of CSVT are highly variable depending on age and underlying acute or chronic illness (3, 7–9). In many cases, the diagnosis may be challenging because symptoms are non-specific and overlap with symptoms of the underlying illness. Neonates present with depressed mental status and seizures. Children with CSVT typically have a triad of symptoms that include depressed mental status, headache, and vomiting, which evolve in an unremitting and progressive manner over a period of days. Mental status changes are variable, and may involve only irritability and drowsiness, or may progress to stupor and coma. Seizures are common, especially in neonates and in children with depressed mental status, and may require video EEG monitoring in order to fully characterize seizure burden and guide anticonvulsant therapy. Physical exam findings may be limited to alterations in mental status, or may include signs of intracranial hypertension such as papilledema and sixth nerve palsy, or a bulging fontanelle in the newborn. Additional signs and symptoms will reflect the underlying provoking illness, such as meningismus in the case of meningitis, or mastoid region tenderness and swelling in the case of mastoiditis. Cavernous sinus thrombosis presents as a distinct clinical syndrome, classically involving a combination of proptosis and chemosis of the involved eye, oculomotor palsies involving any combination of cranial nerves 3, 4, and 6, and sensory loss of the first division of the trigeminal nerve. Cavernous sinus thrombosis typically develops in the setting of infections involving the maxillary and ethmoid sinuses, or as an extension of mastoiditis, and has unique imaging requirements to make the diagnosis (see discussion of imaging in the Section “Diagnostic Approach: Update on Imaging Options and Best Practices”). Children with CSVT whose course is complicated by venous infarction or hemorrhage typically develop seizures and localizing deficits on examination such as hemiparesis, corresponding to the site of the infarction or hemorrhage. In the most severe cases, venous infarction with or without hemorrhage, combined with venous outflow obstruction, may lead to malignant intracranial hypertension, herniation, and death. In children who survive, uncontrolled intracranial hypertension and papilledema may progress to vision loss.

## Diagnostic Approach: Update on Imaging Options and Best Practices

Timely diagnosis and treatment are critically important for optimizing outcome. As in arterial ischemic stroke, “time is brain” should be the guiding principle in managing CSVT. This begins with raising awareness of the clinical signs and symptoms, particularly among front line providers, for children who are at greatest risk—neonates, children with acute head/neck infections, and children with those chronic diseases carrying an increased risk of thromboembolism. These high-risk chronic diseases include those with congenital heart disease, nephrotic syndrome, immunologic disorders, anemia, and leukemia. The triad of symptoms—progressive unremitting headache, altered mental status, and vomiting—should prompt consideration of a diagnosis of CSVT, and to neuroimaging evaluation specifically targeting this condition. Neurologic consultation and direct dialog with radiologists are important strategies to determine the best modality and timing of imaging so as to guide treatment decisions in a timely way.

A variety of imaging modalities can be used. Magnetic resonance imaging (MRI) and MR venography offer the most detailed and sensitive means to assess the clot burden and extent of parenchymal injury. Greater availability of MRI, and improved quality of imaging, particularly with higher strength magnets, means that non-invasive imaging has largely replaced the catheter angiography for the diagnosis of CSVT. Computed tomography (CT) and CT venography provide generally high sensitivity for identifying thrombosis, but are less specific and less sensitive for characterizing brain injury. For example, Roland et al. found that non-enhanced CT has a 73% sensitivity for correctly identifying CSVT, with a very low rate of false positives (15). CT offers the advantage of greater accessibility and speed of imaging, but involves exposure to ionizing radiation and contrast, which is of particular concern in the pediatric population. MRI often is less readily available and requires more support from anesthesia/critical care to manage sedation. Radiologic confirmation of a diagnosis of cavernous sinus thrombosis has distinct requirements. It is best evaluated with contrast-enhanced brain MRI and will be missed by non-enhanced CT and by non-enhanced MRI and conventional MR venography (16).

Specific choices for imaging in any given case often depend on ease of access, time sensitivity for starting treatment, and the range of treatment decisions to be made. For example, a child with acute infectious mastoiditis who has intact mental status and only complains of headache will have treatment decisions involving possible surgical interventions that take priority over starting anticoagulation (AC). In such a case, obtaining the most detailed anatomical information about the brain and the parameningeal structures is most important for planning both the surgical and the antithrombotic treatment. MRI with and without contrast, and MRV, are most suitable in such a case. Contrasting this scenario is that of the child with inflammatory bowel disease who develops rapid and severe declining mental status and focal seizures during a disease flare. In cases like this, where time is of the essence, and sedation for lengthy MRI examination may proves practically challenging, then CT with CT venogram can provide the necessary data to confirm the diagnosis and to make rapid treatment decisions about antithrombotic therapy.

## Treatment: Updates on Treatment Guidelines and Current Practices

There have been no clinical trials evaluating the risks and benefits of treatments for CSVT in children. Published treatment guidelines for children have largely been extrapolated from data obtained from adult studies (17–19). Treatment guidelines for adults recommend the following approach:

(1) Evaluation and treatment of patients with CSVT in facilities with specialized cerebrovascular expertise is appropriate and may be beneficial.
(2) Treatment with AC is safe and may be beneficial for reducing mortality and long-term morbidity, even in the presence of intracranial hemorrhage (ICH).
(3) There is insufficient evidence to show whether heparin or low molecular weight heparin (LMWH) is superior.
(4) The use of fibrinolytic therapy or endovascular therapy may be life-saving in critically ill patients experiencing clinical deterioration despite treatment with AC.
(5) Addition of aspirin or steroids is not recommended due to an association with higher rates of mortality and poor outcome.
(6) Duration of AC treatment: 3–6 months is a reasonable duration of treatment for patients with provoked CSVT; 6–12 months for patients with spontaneous unprovoked CSVT in the absence of a strong permanent thrombophilia; lifelong for patients with a severe thrombophilia (severe genetic deficiency of protein C or S or antithrombin III, homozygous prothrombin or factor V Leiden mutation, antiphospholipid antibody syndrome).

Management guidelines specific to neonatal and pediatric populations have been published, but are limited due to low quality of evidence (14, 20–22). A number of controversies persist, emphasizing the need for more research. As regard the role of thrombophilia testing, there are data in pediatric populations showing that thrombophilias may increase the risk of incident and recurrent CSVT, but it is unknown whether prolonging the duration of AC therapy due to the presence of these conditions alters outcomes (12, 23). As such, the utility of extensive testing for thrombophilia in neonates and children remains uncertain, and deserves further study.

Major uncertainty and controversy exists regarding the treatment of neonates with CSVT. The data from descriptive cohort studies suggest that outcomes are worse among neonates compared to children with CSVT (see Table 2). Moreover, in one observational study of neonates, there was a significant incidence of clot propagation and related new infarction in neonates who were not treated with AC (5). Current data suggest that AC therapy in children and newborns is generally safe, but the efficacy is not established (5, 9). Overall recurrence rates are low (<10%) and may be increased in patients not initially treated with AC therapy (23). Existing cohort studies show that AC therapy is less widely practiced for neonates as compared to children (Table 2). The less frequent use of AC therapy in neonates likely reflects uncertainty about its safety because of the relatively common occurrence of intracranial bleeding from the birth process, and because of the propensity for newborns to develop hemorrhagic infarcts. Currently, a clinical trial proposal is being developed to evaluate the safety and efficacy of AC therapy in neonates with CSVT (6).

Existing guidelines for managing neonatal and pediatric CSVT reflect these controversies and uncertainties, and are summarized as follows:

(1) American Heart Association Scientific Statement (20): for neonates, consider AC therapy using unfractionated heparin (UFH) or LMWH in cases with prothrombotic disorders, multiple cerebral or systemic thrombi, or a propagating cerebral thrombus after treatment with just supportive measures. For children, it is reasonable to treat with UFH or LMWH in all cases, whether or not there is secondary ICH. Thrombolysis is not recommended in neonates, but may be considered in children.
(2) American College of Chest Physicians (14): for neonates without ICH, consider AC therapy with UFH or LMWH, and treat for 6 weeks to 3 months. For neonates with ICH, treat with AC therapy (UFH or LMWH) initially or postpone treatment until repeat imaging after 5–7 days shows clot propagation. For children with CSVT without ICH, treat with AC therapy (UFH or LMWH), and transition to warfarin if desired for a minimum period of 3 months. Consider longer duration of AC therapy in children with incomplete recanalization or ongoing symptoms. For children with CSVT in the presence of ICH, treat with AC initially or postpone treatment until repeat imaging after 5–7 days shows clot propagation. Thrombolysis or thrombectomy may be considered in children who have severe CSVT and are not responding to AC therapy.
(3) British Committee for Standards in Haemotology (21): these recommendations apply to all age groups. Children with CSVT and no ICH should receive AC therapy with either LMWH or UFH, and continued for a minimum of 3 months in cases of reversible provoking illness (e.g., infection), for 6 months in the absence of provoking illness, and for longer periods of time in patients with a long-lasting or genetic risk factor or with persistent symptomatic venous outflow obstruction. They also recommend that repeat imaging should be considered prior to stopping AC therapy for all patients who have ongoing symptoms referable to venous thrombosis and in patients where assessing extent of recanalization may change decisions about duration of therapy. In children with significant and symptomatic ICH at the time of diagnosis, they recommend it may be reasonable to withhold AC therapy and to repeat imaging at a short interval to evaluate for clot propagation. Those patients with minimal or asymptomatic ICH may be considered for treatment with AC therapy.

Endovascular therapy for CSVT has received increased attention in recent years, as it has attained wide acceptance and greater availability for the treatment of acute arterial ischemic stroke. Several case series have been published reporting results for this therapy in children with CSVT. Mortimer et al. described results of treating 9 children, age 18 months to 16 years, with endovascular therapies that included catheter-directed thrombolysis, balloon angioplasty, and thrombectomy with the Penumbra device (24). These children were critically ill, mostly comatose, and 8/9 had progressed while on systemic AC therapy. Treatment was successful in achieving partial recanalization in 8/9 cases, none with bleeding complications, and followed by clinical improvement in 8/9 cases. One child died (the one in whom recanalization could not be achieved), and all others survived, with good functional outcomes. Mallick et al. reported that 4 patients, age 18 months to 11 years, among their consecutive cohort of 21 children with CSVT, received catheter-directed thrombolysis for clinical deterioration despite prior treatment with systemic AC therapy (2). Thrombolytic therapy led to full or partial recanalization and clinical improvement in 3 of these cases, while the fourth child died from malignant intracranial hypertension due to extensive treatment-resistant thrombosis. Procedure-related complications occurred in two patients. Waugh et al. described their experience using endovascular catheter-directed infusions of thrombolytic agents in six children with severe life-threatening and progressive CSVT (25). Four of their six patients survived and appeared to benefit, even in the presence of prethrombolytic ICH.

These small case series suggest that endovascular therapy may be helpful in selecting pediatric patients with severely symptomatic, treatment-resistant CSVT. Use of this therapy in children as reflected in these reports is in line with the treatment guidelines proposed for adults. As is the case for any invasive, potentially risky therapy, it would be prudent for providers to account for several factors when considering this therapy for children. First, the potential for procedural complications, and the generally good outcome from standard AC therapy, suggest that endovascular therapy should be reserved for children with severe disease who have failed frontline AC therapy. Second, the procedural risks are likely operator dependent, and so all attempts should be made to involve interventionalists who are experienced in the treatment of children, and that such treatment should occur in a tertiary pediatric center with adequate subspecialty expertise (critical care, anesthesia, vascular neurology, hematology). This is another area where research is needed to more fully characterize the potential for benefit relative to risk in good prospectively ascertained and well-characterized cohorts with long-term follow-up data.

## Author Contributions

The author confirms being the sole contributor of this work and approved it for publication.

## Conflict of Interest Statement

The author declares that the research was conducted in the absence of any commercial or financial relationships that could be construed as a potential conflict of interest.
